# Isolation and Characterization of Phenanthrene Degrading Bacteria from Diesel Fuel-Contaminated Antarctic Soils

**DOI:** 10.3389/fmicb.2017.01634

**Published:** 2017-08-28

**Authors:** Alejandro Gran-Scheuch, Edwar Fuentes, Denisse M. Bravo, Juan Cristobal Jiménez, José M. Pérez-Donoso

**Affiliations:** ^1^Bionanotechnology and Microbiology Lab, Center for Bioinformatics and Integrative Biology, Facultad de Ciencias Biologicas, Universidad Andres Bello Santiago, Chile; ^2^Departamento de Química Inorgánica y Analítica, Facultad de Ciencias Químicas y Farmaceuticas, Universidad de Chile Santiago, Chile; ^3^Laboratorio de Microbiología Oral, Facultad de Odontología, Universidad de Chile Santiago, Chile; ^4^Research and Development Laboratory, uBiome Santiago, Chile

**Keywords:** Antarctica, bioremediation, phenanthrene

## Abstract

Antarctica is an attractive target for human exploration and scientific investigation, however the negative effects of human activity on this continent are long lasting and can have serious consequences on the native ecosystem. Various areas of Antarctica have been contaminated with diesel fuel, which contains harmful compounds such as heavy metals and polycyclic aromatic hydrocarbons (PAH). Bioremediation of PAHs by the activity of microorganisms is an ecological, economical, and safe decontamination approach. Since the introduction of foreign organisms into the Antarctica is prohibited, it is key to discover native bacteria that can be used for diesel bioremediation. By following the degradation of the PAH phenanthrene, we isolated 53 PAH metabolizing bacteria from diesel contaminated Antarctic soil samples, with three of these isolates exhibiting a high phenanthrene degrading capacity. In particular, the *Sphingobium xenophagum* D43FB isolate showed the highest phenanthrene degradation ability, generating up to 95% degradation of initial phenanthrene. D43FB can also degrade phenanthrene in the presence of its usual co-pollutant, the heavy metal cadmium, and showed the ability to grow using diesel-fuel as a sole carbon source. Microtiter plate assays and SEM analysis revealed that *S. xenophagum* D43FB exhibits the ability to form biofilms and can directly adhere to phenanthrene crystals. Genome sequencing analysis also revealed the presence of several genes involved in PAH degradation and heavy metal resistance in the D43FB genome. Altogether, these results demonstrate that *S. xenophagum* D43FB shows promising potential for its application in the bioremediation of diesel fuel contaminated-Antarctic ecosystems.

## Introduction

The southernmost continent in Earth, Antarctica, is a unique territory that plays an important role in the earth's climate, and a source of many natural resources. These characteristics have fueled the interest of many countries to explore and investigate this region, leading to the involvement of over 30 nations in scientific research, and the establishment of more than a 100 facilities in the region (Hughes, 2014; Rack, 2015). Additionally to scientific exploration, a growing touristic industry has generated a surge in human activity in Antarctica. An increasing footprint in the shape of anthropogenic pollutants has become an important problem and hazard toward the native Antarctic ecosystem. Importantly, oil spillage due to transport, storage and utilization of fossil fuels has become a serious and persistent threat (Aislabie et al., 2004; Hughes, 2014). The hydrocarbon contamination in Antarctica has profound effects that have been shown to reshape the structure of microbial communities as well as affecting the abundance of small invertebrate organisms (Saul et al., 2005; Thompson et al., 2007; Powell et al., 2010). Oil contamination can generate detrimental changes in soil properties, including modifications in maximum surface temperature, pH, and carbon and nitrogen levels (Aislabie et al., 2004). These physical and chemical changes promote rearrangements in the soil bacterial communities as well, with Proteobacteria from *Burkholderia, Sphingomonas* and specially *Pseudomonas* species heavily dominating over bacteria from Bacteroidetes, Cyanobacteria, Acidobacteria, Actinobacteria, Chloroflexi, Gemmatimonadetes, Verrucomicrobia, and other phyla which compose the normal inhabitants non-polluted Antarctic soils. Altogether, this results in a significant decrease in species richness and evenness, and a large decline in soil biodiversity of contaminated soils (Saul et al., 2005; van Dorst et al., 2014, 2016).

Diesel oil, the most commonly used fuel in Antarctica, contains a variety of toxic compounds that persist in contaminated soils, including heavy metals like Cd, Cr and Pb; and polycyclic aromatic hydrocarbons (PAH) like naphthalene, phenanthrene, and fluorene (Lee et al., 1992). Microbial bioremediation, the process of degradation of contaminants by the metabolic activities of microorganisms, is an ecological, economical and safe approach that can be applied to the decontamination of PAHs with minor alteration of the soil (Bamforth and Singleton, 2005). This process can be achieved by promoting the growth of endogenous metabolizing bacteria in contaminated sites (biostimulation) or by directly seeding contaminated sites with pollutant-degrading bacteria (bioaugmentation). Since the Antarctic Treaty impedes the introduction of foreign organisms into the Antarctic continent, bioaugmentation can only be implemented by the use of native microbes. Interestingly, this method is the best choice in the bioremediation of soils with low indigenous PAH-degrading bacteria (Castiglione et al., 2016). Because of this, the study and isolation of novel native PAH-degrading bacteria constitutes an approach with tremendous potential, and a powerful alternative for biotechnological treatments of not age-old PAHs-contaminated soils, such as bioaugmentation, *ex situ* treatment or for emerging methods such as enzyme-mediated bioremediation (Kuppusamy et al., 2017), specially in the case of Antarctic remediation. Various hydrocarbon-degrading bacteria have been isolated to date from Antarctica, including *Rhodococcus, Acinetobacter, Pseudomonas*, and *Sphingomonas* species (Aislabie et al., 2006). The use of serial PAH-supplemented growth media enrichments, and the highly selective growth condition of utilizing PAHs as sole energy source, have permitted to establish successful approaches to isolate nalovel PAH-degrading bacteria of interest from varied environmental sources (Obi et al., 2016; Alegbeleye et al., 2017; Li et al., 2017; Oyehan and Al-Thukair, 2017). Additionally, new techniques of *in situ* microculturing have allowed to culture an even broader number of PAH-degrading organisms by recreating the native conditions of growth, allowing growth of bacteria not normally isolated using standard culture conditions (van Dorst et al., 2016). Together with the capacity to grow environmental strains, the ability to screen the large number of growing bacteria for those isolates possessing a high PAH-degrading ability is essential to find the most effective bacterial strains for their use into successful bioremediation strategies. A common problem of the determination of PAHs in complex matrices is the diversity of potential interferences and the low analyte levels (Yusty and Davina, 2005). The method more frequently used to determine bacterial degradation of PAHs is the extraction and purification of lipophilic compounds followed by chromatographic determination (Schuler et al., 2009; Madueño et al., 2011; Kuppusamy et al., 2016). This methodology is time consuming, laborious and expensive, due to the sample pretreatment and the subsequent analytical determination. However, PAHs, including phenanthrene, are naturally fluorescent and detectable by fluorescence spectroscopy as alternative to chromatographic analysis, allowing to perform rapid and inexpensive measurements (Vasquez et al., 2013). Moreover, a described alternative to improve the selectivity of this analytical method is the use of chemometric tools that allow the determination of analyte concentrations and the estimation of spectral profiles of sample components in the presence of many unknown constituents (Alarcon et al., 2013; Vasquez et al., 2013).

Diesel-fuel contains high concentrations of two- and three-ringed PAHs, including phenanthrene. Since it exhibits higher solubility than others PAHs in water and its degradation products generate changes in the coloration of culture media (Treccani, 1965; Lee et al., 1992), phenanthrene is an attractive compound to enrich for and to follow PAH degradation by environmental isolates. Additionally, phenanthrene is the smallest PAH to present both Bay-region and K-region conformations, that allow the formation of different reactive species, making it a model substrate for PAH metabolism (Samanta et al., 2002). In this work, we isolated PAH degrading bacteria from Antarctic soil samples collected during the 49th Scientific Antarctic Expedition to the South Shetland Islands. Samples were collected from non-diesel-exposed and diesel fuel-contaminated sites, and bacteria of interest were isolated by a three-step enrichment and screening approach, based on their ability to metabolize phenanthrene. Three isolates were chosen for further characterization based on their high phenanthrene degradation ability, and show a promising potential for their application in the bioremediation of diesel fuel contaminated-Antarctic ecosystems.

## Materials and methods

### Sample collection

Antarctic soil samples were collected from sites across King George Island, located in the South Shetland Islands (62°10 S, 58°49 W) during the 49th Scientific Antarctic Expedition (ECA 49) during February 2013. Samples were taken from the surface soil horizon (0–10 cm) from four sites exposed to diesel fuel, and four non diesel-exposed control sites, to collect the samples were used spatulas cleaned with ethanol 70% v/v and demineralized water, prior the collection the spatula was in contact with the soil near the collection point. Samples were sealed in sterile tubes and transported on ice to the laboratory and analyzed after 2 months.

### Isolation of phenanthrene degrading bacteria

A three-step enrichment and screening process was followed to isolate high-metabolizers of phenanthrene from soil samples. Aliquots from four diesel fuel-exposed soils and four non-exposed control soils were resuspended in sterile distilled water (50% w/v), supplemented with UV-light sterilized phenanthrene (20% w/v), and incubated 72 h at 8, 18, and 28°C. Samples were then plated in Reasoner's 2 (R2A) agar and incubated at 8, 18, and 28°C until colony growth was observed. A total of 350 different colonies were picked, grown in R2A medium, washed twice with 25 mM phosphate buffer, and used to inoculate separate tubes containing M9 minimal media with 2,000 ppm of phenanthrene as sole carbon source. Cultures were grown with agitation at room temperature for 5 days, and phenanthrene metabolizing strains screened by change in medium color from clear to yellow due to the generation 2′-hydroxy muconic semialdehyde, a degradation product of phenanthrene (Treccani, 1965). 53 cultures that metabolized phenanthrene were selected, and the concentration of PAH quantified as described below. The three highest metabolizing strains were then selected for further studies.

### Phenanthrene quantification

To quantify three-ring PAH concentrations, excitation-emission fluorescence spectroscopy with multivariate data analysis was followed (Alarcon et al., 2013). Briefly, non-polar compounds were extracted from culture media using two volumes of hexane and vigorous mixing for 60s. Extraction was repeated three times. The excitation-emission spectra of each sample were measured in a Varian Cary-Eclipse luminescence spectrometer (Mulgrave, Australia), and PAH concentration was determined by means of second order calibration using the PARAFAC and U-PLS/RBL algorithms. All chemometric computation and routines were implemented in Matlab v.7.6 (Mathworks, Natwick, MA).

### Bacterial strains and culture media

Environmental bacterial isolates, as well as the control strains *Escherichia coli* BW25113 and *Pseudomonas aeruginosa* PAO1, were routinely grown in R2A and M9 minimal media. Single colonies were picked and grown overnight in liquid medium with agitation at 28°C. The cultures were diluted into fresh medium 1:100, and grown until they reached an OD_600_ = 0.1. For solid media preparation, 2% w/v of agar was added to R2A and M9 media.

### Identification of environmental bacterial isolates

Primers 27F (AGAGTTTGATCMTGGCTCAG) and 1492R (TACGGYTACCTTGTTACGACTT) were used to amplify the 16s gene by PCR. After analyzing obtained products by agarose gel electrophoresis, DNA samples were purified, Sanger sequenced, and obtained sequences queried against NCBI 16S databases. Sequences have been deposited online, and available through the GenBank database with the following accession numbers: *R. erythropolis* D32AFA: MF407275; *Sphingobium xenophagum* D43FB: MF406207; and *P. guineae* E43FB: MF407316.

### Bacterial analyses

The analytical profile index of isolates was analyzed with a commercial kit, following manufacturer's instructions (API 20 NE, BioMerieux). The optimal growth temperature was determined by a previously published protocol (Gallardo et al., 2014). The production of biosurfactants was assessed by measuring the E_24_ index following a published protocol (Wei et al., 2005). Briefly, bacterial strains were grown in M9 media with phenanthrene as sole carbon source, and after 5 days of growth at 28°C, cells pelleted by centrifugation, supernatants recovered and mixed with diesel oil. After vigorous shaking, samples were incubated 24 h, and the E_24_ index estimated as the percentile of the height of the emulsion vs. the height of the culture. Surfactant production was also assessed by a droplet collapse experiment. 5 μL of concentrated supernatants were mixed with 2 μL of methylene blue solution, and collapse of droplet in a flat surface estimated. For both experiments water was used as a negative control, and 0.5% SDS as a positive control. Chemotaxis was assessed using a modified capillary assay (Gordillo et al., 2007) that measures the ability of bacteria to swim through a syringe needle toward a solution with a target compound. Bacteria were grown in R2A medium, pelleted, and washed with chemotaxis buffer (Tris-HCl 10 mM, pH 7.4). 5 × 10^7^ CFU/mL of bacterial suspension were collected in a pippete tip, and connected through a needle to a sterile syringe containing chemotaxis buffer plus chemoatractant substances (glucose, phenanthrene). After 90 min incubation at room temperature, buffer contained in the syringe was recovered, serially diluted, and plated to estimate CFU/mL.

### Biofilm formation assays

Biofilm production was assessed by a published protocol (O'Toole, 2011). Bacterial isolates were grown to an OD_600_ = 0.1, and then diluted 1:100 with fresh M9 media into 96 well polystyrene plates. After 24 h incubation, growth was estimated by measuring the absorbance at 600 nm. Afterwards, media was aspirated, wells washed and stained with a crystal violet solution. The dye was extracted using an ethanol-acetone solution, and absorbance at 590 nm measured. The absorbance of the crystal violet was then normalized to the optical density of each culture.

### Phenanthrene adhesion assay

Overnight bacterial cultures were back-diluted 1:1,000 into fresh M9 media with 2,000 mg/L sterile phenanthrene as sole carbon source. After 5 d of incubation at 28°C, phenanthrene crystals were washed, stained with a crystal violet solution, and analyzed by light microscopy.

### Growth kinetics using phenanthrene or diesel-fuel as sole carbon sources

Overnight cultures grown in R2A medium were centrifuged 5 min at 7,000 RPM, and washed twice with 25 mM phosphate buffer. Strains were then diluted 1:1,000 into fresh M9 media with sterile phenanthrene (0.05%) or diesel-fuel (0.2%) as sole carbon sources. Cultures were grown at 28°C with agitation for 168 h, and every 24 h samples were taken, serially diluted and plated to determine viable colony forming units (CFU). *E. coli* BW25113 or PAO1 were used as negative control. To measure the effect of cadmium over growth with phenanthrene as carbon source, increasing concentrations of CdCl_2_(0, 0.025, 0.05, 0.5, and 5 mg/L)were added to culture tubes. PAH concentrations were measured as stated above.

### Scanning electronic microscopy

To analyze cell adhesion to phenanthrene crystals, a bacterial culture was grown in M9 media with phenanthrene as sole carbon source. After 24 and 120 h of growth at 28°C, an aliquot was taken from the culture and transferred to a copper grid. After air-drying, and without further coating treatments, the samples were visualized in a LVEM5 scanning electron microscope using 5 kV.

### Whole genome sequencing

A genomic DNA library of *S. xenophagum* D43FB gDNA was constructed and sequenced using the Illumina MiSeq platform. We designed a 540-bp paired-end library to generate paired-end sequencing reads of 2 × 300 bp. Then, sequenced reads were trimmed based on their quality scores (Q ≥ 30). A *de novo* genomic assembly was performed using CLC Genomics Workbench version 6.5.2 (length fraction, 0.5; similarity fraction, 0.9) and SOAP *de novo* version 1.05. This genome has been submitted to NCBI for online access (Bioproject PRJNA392681). Similarity searches were performed to assign D43FB genes to PAH degradation pathways, based on information provided in KEGG website (http://www.kegg.jp/). Chemical structures used to build degradation pathway scheme in **Figure 5** taken with permission from KEGG website (Kanehisa and Goto, 2000).

### Statistical analyses

All analyses were performed using GraphPad Prism v5.0 software. To assess significant differences between more than two groups of data, one-way ANOVA test was used, with the Tukey post-test used to compare each different group, using a *p* < 0.05.

## Results

### Isolation of phenanthrene degrading strains from antarctic soil samples

**Figure 1:**
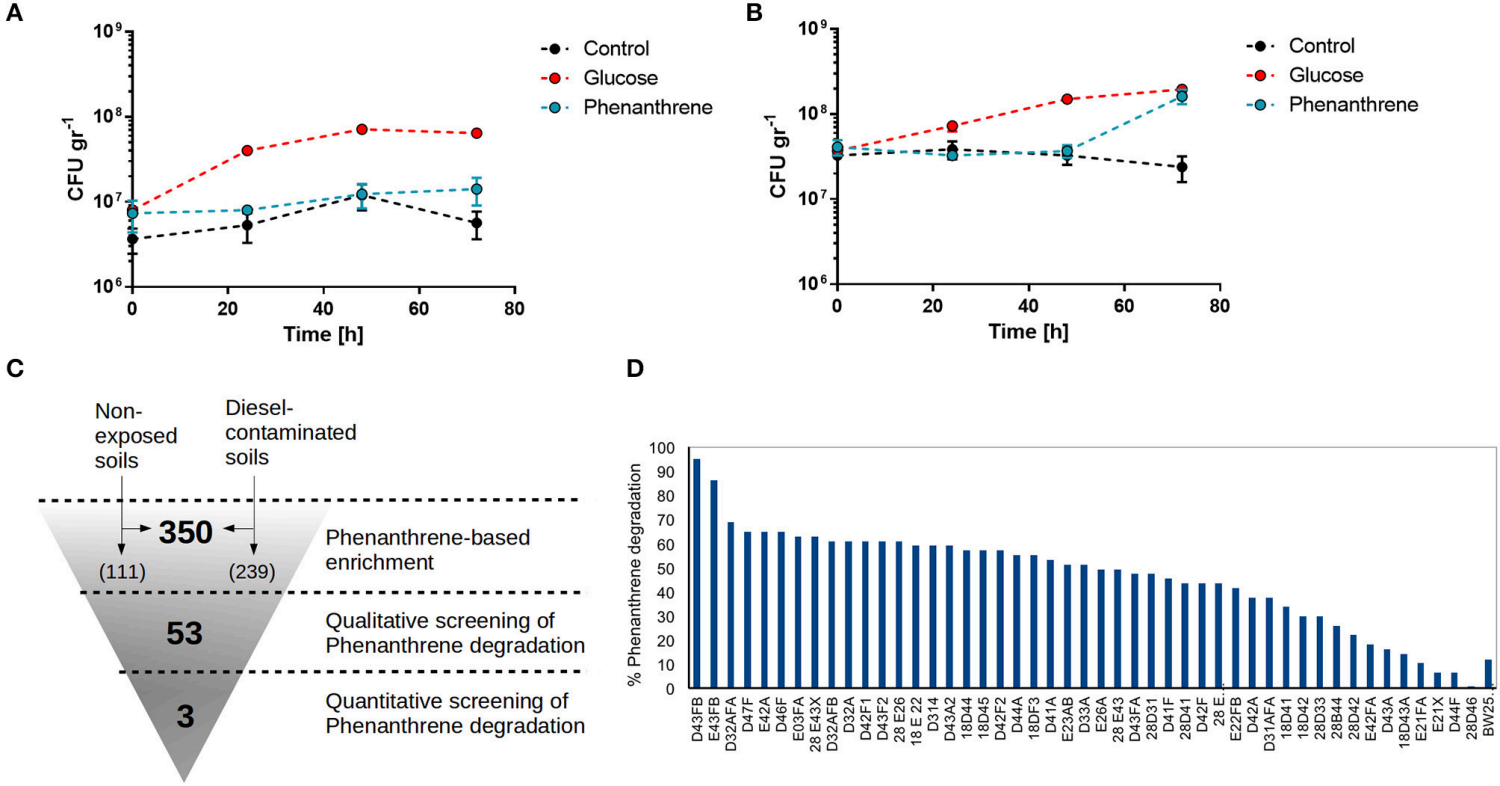
Isolation of phenanthrene degrading bacteria from Antarctic soils. **(A,B)**. Antarctic soil samples from unexposed **(A)** and diesel-fuel exposed sites **(B)** were assessed for bacterial growth without supplementation, or after supplementation with 0.2% glucose or 0.2% phenanthrene. **(C)** Workflow for isolation of phenanthrene degrading strains from Antarctic soils. **(D)** Percentage of phenanthrene degradation by isolated Antarctic strains.

To first test the ability of phenanthrene to enrich for PAH degrading bacteria, total bacterial growth in Antarctic soil samples was tested when supplemented with glucose or phenanthrene and compared with a non-supplemented control. Soil samples from non-contaminated as well as diesel-exposed sites were used. Bacterial growth in soil from non-contaminated sites was promoted when glucose was added, but not in control conditions or when phenanthrene was present (Figure 1A). In contrast phenanthrene supplementation of a diesel-exposed soil promotes sudden bacterial growth after 72 h, suggesting that phenanthrene is being metabolized and used as a carbon source by the bacteria present in contaminated soils (Figure 1B). Glucose supplementation and control condition for the diesel-exposed soil showed similar growth behavior as non-exposed soil. Next, we proceeded to isolate phenanthrene-metabolizing bacteria from selected soils samples by a three-step enrichment and screening process (Section Materials and Methods, Figure 1C). After an initial enrichment of PAH-degrading bacteria by phenanthrene supplementation of soil samples, a total of 350 colonies were isolated, 111 originating from non-diesel-exposed sites and 239 originating from diesel-contaminated soils. These 350 colonies were then screened for phenanthrene degradation, by qualitatively assessing the change in medium color generated by phenanthrene degradation products (Treccani, 1965). After this first screening, 53 colonies were chosen to undergo a quantitative screening, in which the concentrations of phenanthrene in culture media were measured by fluorescence spectroscopy. Six strains generated an unexpected higher level of fluorescence at the end of the experiment compared with initial levels of phenanthrene fluorescence, and were thus excluded from further analyses. The remaining 47 strains exhibited a range of phenanthrene degradation capacities, with 24 strains degrading more than 50% of initial phenanthrene (Figure 1D). The three highest phenanthrene-metabolizing strains were all isolated from diesel contaminated soils, and corresponded to strains D43FB (95% degradation), E43FB (86% degradation), and D43AFA (69% degradation). These three strains were chosen to undergo further characterizations of their bioremediation potential.

### Characterization of phenanthrene degrading strains

**Figure 2:**
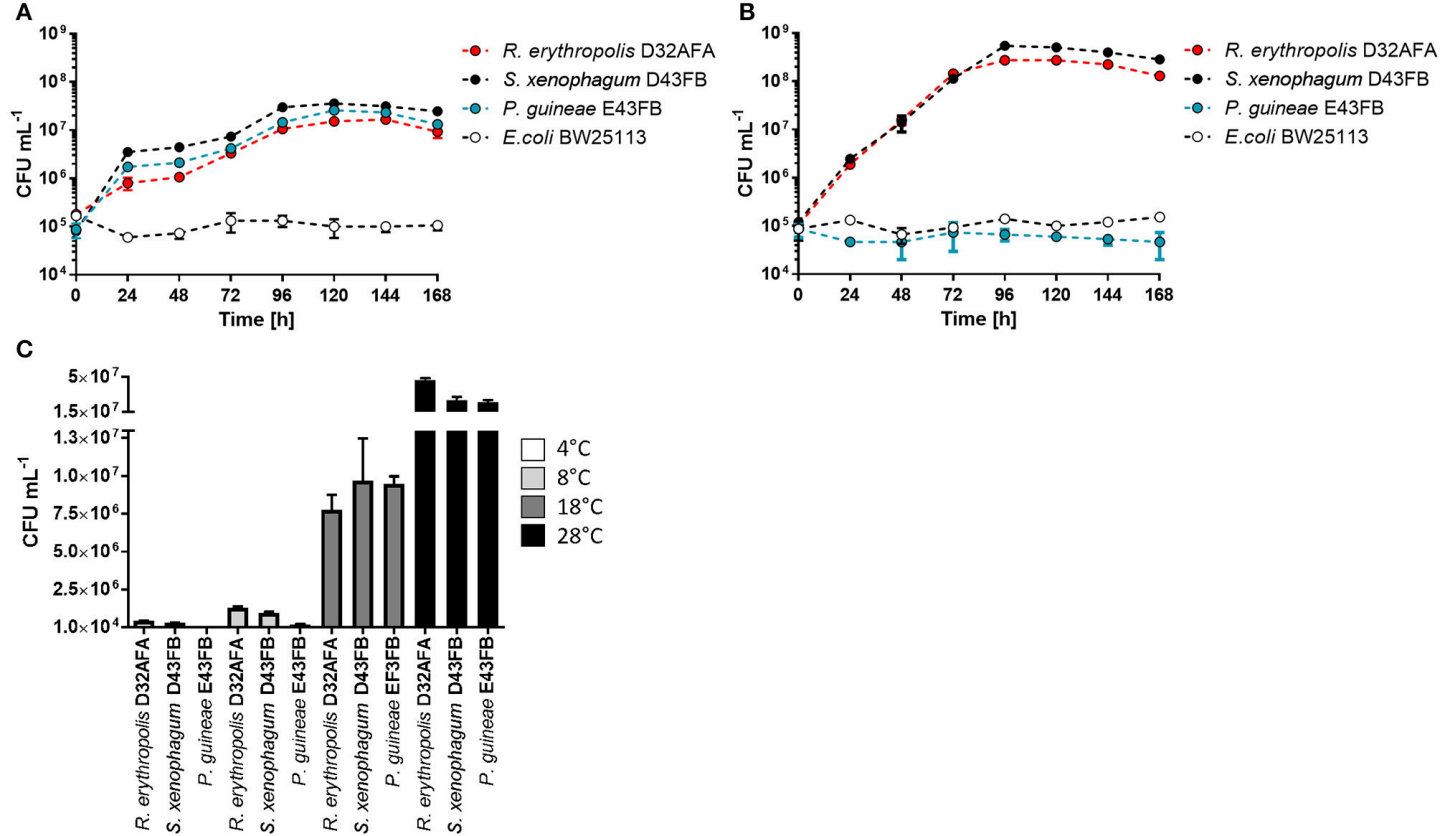
Growth characterization of phenanthrene degrading strains. **(A)** Growth of isolated strains in liquid M9 medium, with 0.05% phenanthrene as sole carbon source. **(B)** Growth of isolated strains in liquid M9 medium, with 0.2% diesel-fuel as sole carbon source. **(C)** Determination of optimal growth temperatures for isolated strains.

D32AFA, D43FB, and E43FB were identified by 16S rDNA sequencing, which revealed these strains corresponded to isolates of *Rhodococcus erythropolis, S. xenophagum*, and *Pseudomonas guineae*, respectively. We first characterized the growth kinetics of the three isolates using phenanthrene as sole carbon source (Figure 2A). The three Antarctic strains exhibit a long lag phase, and after 48 h start rapidly proliferating, with *S. xenophagum* D43FB exhibiting the highest growth yield. *E. coli* BW25113, included as a negative control, is unable to use phenanthrene as an energy source and does not proliferate under these growth conditions. When the experiment was repeated using diesel-fuel as sole carbon source (Figure 2B), *R. erythropolis* D32AFA and *S. xenophagum* D43FB showed a similar growth pattern, with ten-fold higher numbers of bacterial cells compared to growth in phenanthrene. *P. guineae* E43FB on the other hand was unable to use diesel fuel as energy source. We also tested the Antarctic strains on a metabolic panel of carbon sources (Table S1). The phenotypic characterization showed positive utilization of substrate for the three stains for D-glucose, L-arabinose and D-maltose. Additionally, *R. erythropolis* D32AFA and *S. xenophagum* D43FB can grow using a variety of compounds as energy source, like D-mannose, D-mannitol, capric acid, malic acid and citrate, while *P. guineae* E43FB proved more selective in its carbon-source requirements. Analysis of optimal growth temperature revealed that all isolates exhibit maximum growth rate at 28°C (Figure 2C), indicating that these strains are psychrotolerant rather than psychrophylic, a behavior we have seen in previously isolated Antarctic strains (Gallardo et al., 2014; Órdenes-Aenishanslins et al., 2016; Plaza et al., 2016).

### Phenanthrene-degrading strains can form biofilms and adhere to phenanthrene crystals

**Figure 3:**
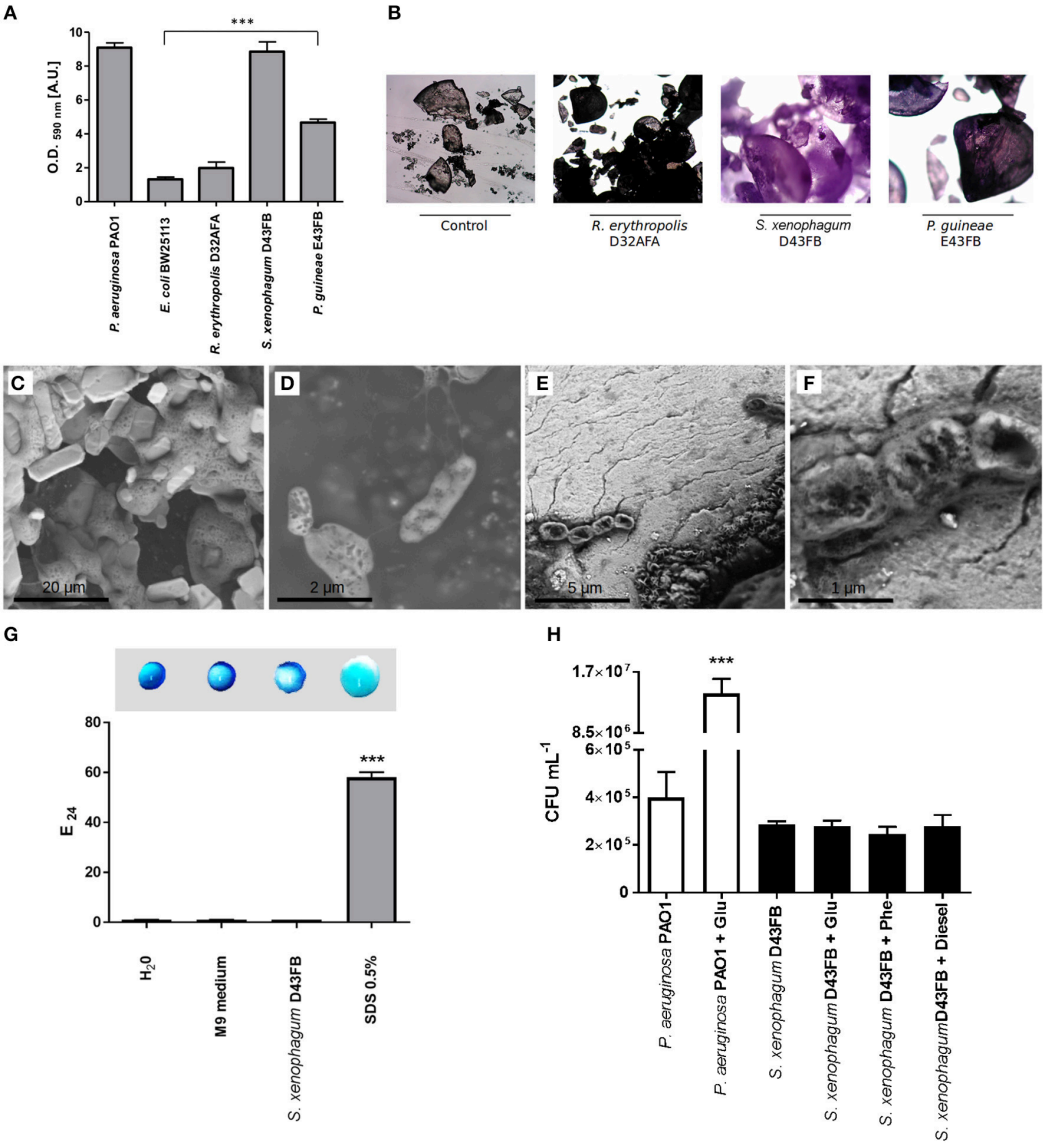
Biofilm production, biosurfactant and chemotactic responses of phenanthrene degrading isolates. **(A)** Biofilm production on polystyrene microtiter plates. Adhered biomass was quantified by crystal violet staining. *P. aeruginosa* PAO1 and *E. coli* BW25113 were used as controls. Values of Antarctic strains were compared to *E. coli* to establish statistical differences. **(B)** Adhesion of bacteria to phenanthrene crystals. Crystals were incubated with degrading strains, washed, stained with crystal violet and examined by light microscopy. **(C–F)** The interaction between strain D43FB and phenanthrene was studied by scanning electron microscopy. **(C)** Phenanthrene crystals. **(D)** *S. xenophagum* D43FB grown with glucose as sole carbon source. **(D,E)** *S. xenophagum* D43FB grown with phenanthrene as sole carbon source. **(F)** Biosurfactant production was assessed by droplet collapse (top) and E24 index (bottom), using spent media from *S. xenophagum* D43FB growth. Water and M9 media were used as negative controls, and 0.5% SDS used as positive control. **(G)** Chemotaxis by *P. aeruginosa* PAO1 and *S. xenophagum* D43FB toward different compounds was assessed using a modified capillary assay. **(H)** Number of bacterial cells that were attracted toward glucose, phenanthrene, and diesel-fuel were assessed after incubation, and compared toward buffer only control condition. ^***^ = Statistical significance with *p* < 0.001.

One mechanism shown to promote the ability of bacteria to degrade PAHs it's the direct attachment to the surface of hydrocarbon compounds, often in the form of biofilm communities (Eriksson et al., 2002; Rodrigues et al., 2005). To test the adherence capabilities of selected strains, we first tested their ability to form biofilm in polystyrene microtiter plates (Figure 3A). *S. xenophagum* D43FB and *P. guineae* E43FB were able to generate robust biofilm growth in comparable levels to the control strain *P. aeruginosa*. On the other hand, *R. erythropolis* D32AFA was unable to generate biofilms. We then proceeded to analyze the ability of selected isolates to adhere to phenanthrene crystals. After incubation with bacteria, phenanthrene crystals were washed and stained with crystal violet. As shown in Figure 3B, crystals incubated with *S. xenophagum* D43FB and *P. guineae* E43FB exhibited a strong and intermediate staining, respectively. Staining of crystals incubated with *R. erythropolis* D32AFA did not differ from the control, suggesting this strain is unable to adhere to phenanthrene. To further study the ability of *S. xenophagum* D43FB to bind phenanthrene, incubated crystals were analyzed by scanning electron microscopy (SEM; Figures 3C–F). Control strains grown in presence of glucose exhibited rod morphology consistent with *Sphingobium* species (Figure 3D). When phenanthrene crystals incubated with bacteria were analyzed, microscopy analysis revealed how D43FB cells intimately attach to the phenanthrene crystals that serve as its carbon source (Figure 3E), supporting crystal adhesion results.

We sought to understand how *S. xenophagum* D43FB promoted its efficient metabolism of phenanthrene. Besides adhesion and biofilm formation, other mechanisms have been shown to enhance the ability of bacteria to metabolize PAHs, including the secretion of biosurfactants that increase the bioavailability of aromatic compounds (Volkering et al., 1998). We first tested the ability of D43FB to release biosurfactants to culture medium. To this end, we assessed the ability of culture supernatants to emulsify diesel (E_24_ index) as well as the ability of supernatants to generate water droplet collapse by altering surface tension (Figure 3G). In both tests, D43FB supernatants lacked emulsifying abilities, and exhibited similar behaviors to negative controls, suggesting D43FB does not secrete biosurfactants to aid in its degradation of phenanthrene.

Chemotaxis, the ability of bacteria to modulate their swimming responses in response to chemical cues, has also been proposed to contribute to the process of PAH degradation. We studied the *swimming* and *swarming* motility present on *S. xenophagum* D43FB using plates containing M9 media with 0.5% (*swimming*) and 0.25% (*swarming*) agar (not shown). No swimming or swarmming motility was determined. In addition, we evaluated the ability of *S. xenophagum* D43FB to positively respond toward phenanthrene. To this end, we used a modified capillary assay that assesses the ability of bacteria to swim through a syringe needle toward a solution with a target compound (Gordillo et al., 2007). Control strain *P. aeruginosa* was able to accumulate in a syringe containing a glucose solution (Figure 3H). In comparison, *S. xenophagum* D43FB didn't show any increased chemotactic response toward glucose, phenanthrene or diesel when compared with buffer-only control condition, suggesting chemotaxis is not involved in PAH degradation processes in this strain.

### Cadmium impairs *S. xenophagum* D43FB metabolism of phenanthrene

**Figure 4:**
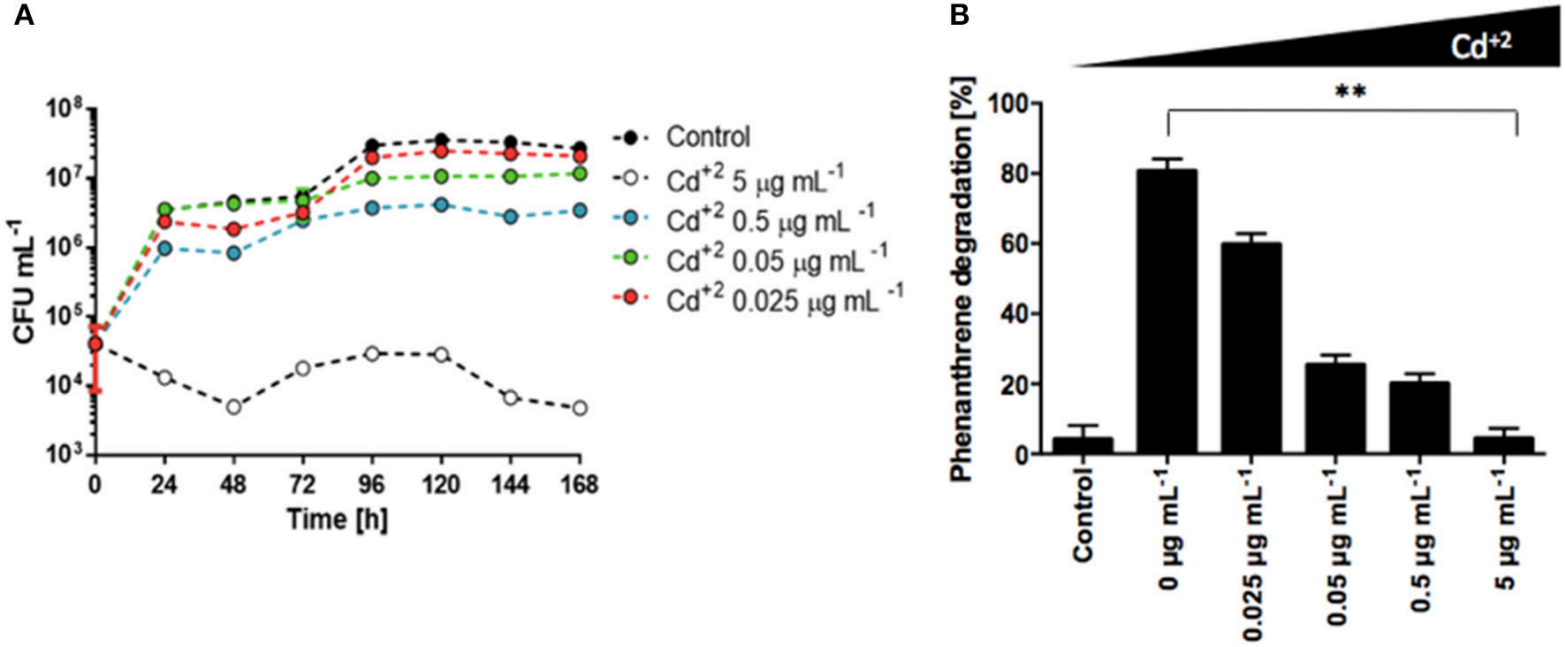
Cadmium impairs phenanthrene degradation by *S. xenophagum* D43FB. Bacterial growth and phenanthrene degradation were assessed in the presence of cadmium. **(A)** *S. xenophagum* D43FB was grown in M9 medium with phenanthrene as sole carbon source and increasing concentrations of CdCl_2._ Viable bacterial cells were assessed during a 7-day period. **(B)** Phenanthrene concentrations were quantified after 7 days of bacterial growth. ^**^ = Statistical significance with *p* < 0.01.

It has been shown that presence of heavy metals can generate changes in the activities of microbial communities, affecting enzymes involved in different metabolic processes, and the microbial degradation of diesel fuel has been shown to be impaired as well in presence of varied heavy metals (Kandeler et al., 1996; Riis et al., 2002). Analysis of the Antarctic soil samples from diesel contaminated sites used in this study revealed high concentrations of heavy metals, with Cd concentrations between 15 and 85 mg/Kg (data not shown), above of what is permitted by several international norms (heavy metal standard of soils for Netherlands is until 12 mg/Kg, for Canada and UK is 10 mg/Kg and for Chile 40 mg/Kg) (Gran-Scheuch et al., under review). To test the effects of cadmium over the growth and metabolism of phenanthrene, we grew *S. xenophagum* D43FB in media containing phenanthrene as sole carbon source, and increasing Cd concentrations. The amount of total bacterial growth decreased when Cd concentrations were increased, with complete growth arrest generated at high concentrations (Figure 4A). Analysis of phenanthrene levels revealed that in the absence of cadmium, incubation with *S. xenophagum* D43FB generated the degradation of almost 80% of the initial phenanthrene (Figure 4B). Increasing Cd concentrations generated a reduction in the ability of D43FB to metabolize phenanthrene, although up to 20% of the initial phenanthrene amount is still metabolized at relatively high Cd concentrations (Figure 4B). These results highlight how *S. xenophagum* D43FB can metabolize phenanthrene even in the presence of toxic heavy metals present in diesel.

### The *S. xenophagum* D43FB genome harbors genes involved in phenanthrene metabolism

**Figure 5:**
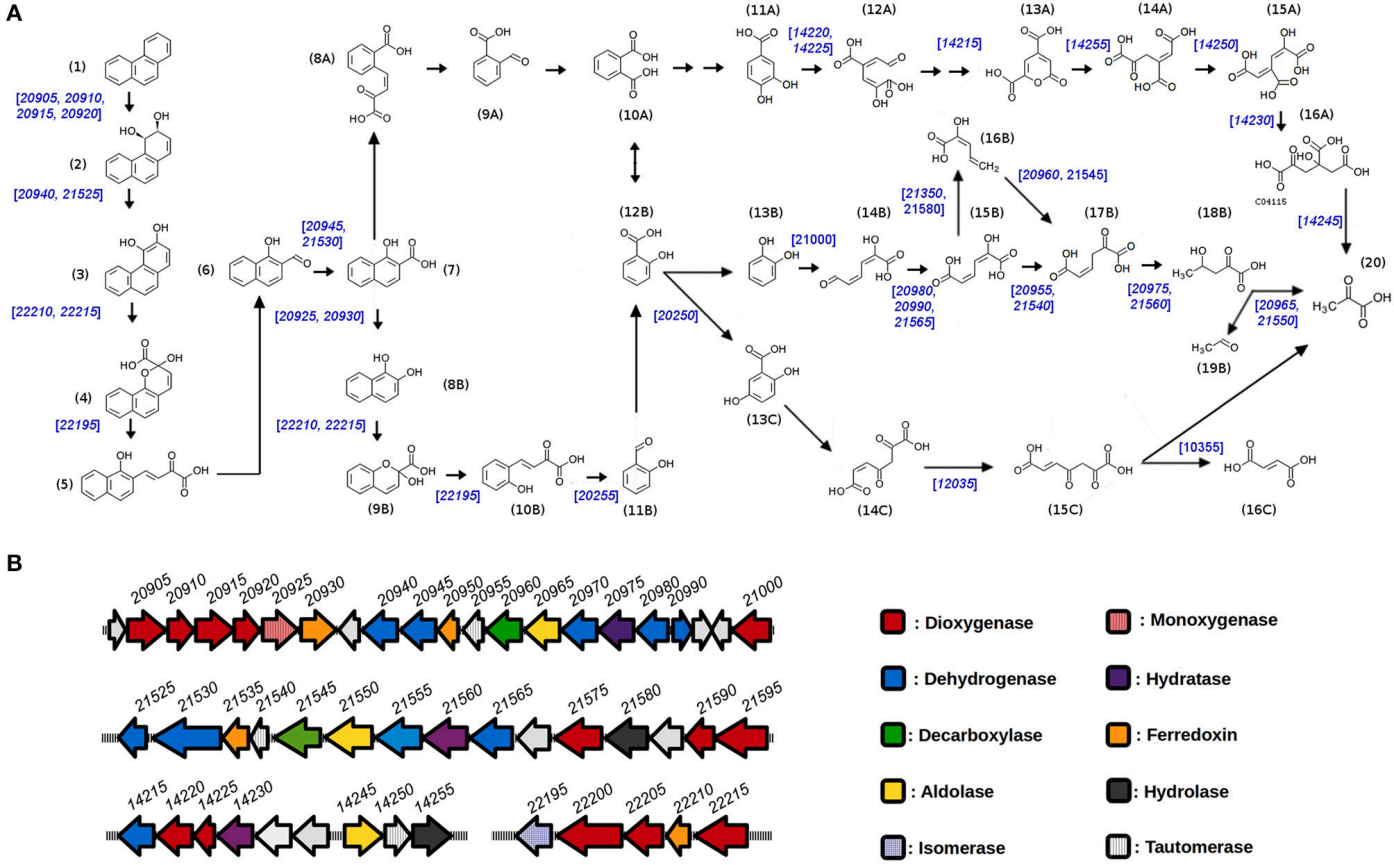
Genetics of Phenanthrene degradation in *S. xenophagum* D43FB. **(A)** Predicted pathways of aerobic Phenanthrene degradation are shown, and genes mapped to D43FB genome with required enzymatic activies are shown in blue numbers. Chemical structures: (1) phenanthrene, (2) *cis-*3,4-Dihydrophenanthrene-3,4-diol, (3) phenanthrene 3,4-diol, (4) 2-hydroxy-2H-benzo[h]chromene-2-carboxylate, (5) *cis*-4-(1′-hydroxynaphth-2′-yl)-2-oxobut-3-enoate, (6) 1-hydroxy-2-naphthaldehyde, (7) 1-hydroxy-2-naphthoate, (8A) *cis*-2′-carboxybenzalpyruvate, (9A) 2-formylbenzoate, (10A) phthalate, (11A) protocatechuate, (12A) 4-carboxy-2-hydroxymuconate semialdehyde, (13A) pyrone-4,6-dicarboxylate, (14A) oxalomesaconate, (15A) 4-carboxy-2-hydroxy-*cis*,*cis*,-muconate, (16A) 4-hydroxy-4-carboxymethyl-2-oxoglutarate, (8B) naphthalene-1,2-diol, (9B) 2-hydroxychromene-2-carboxylate, (10B) *trans*-o-hydroxybenzylidenepyruvate, (11B) salicylaldehyde, (12B) salicylate, (13B) catechol, (14B) hydroxymuconate semialdehyde, (15B) hydroxymuconate, (16B) 2-hydroxy-pentadienoate, (17B) oxalocrotonate, (18B) 4-hydroxy-2-oxovalerate, (19B) acetaldehyde, (13C) gentisate, (14C) maleylpyruvate, (15C) fumarylpyruvate, (16C) fumarate, (20) pyruvate. **(B)** Organization of predicted Phenanthrene degradation genes in *S. xenophagum* D43FB. Principal operons encoding enzymatic functions involved in PAH degradation are shown, with predicted function and gene numbers.

Microbial degradation of phenanthrene can proceed by three different methods: mineralization, co-metabolic transformation and oxidation. In the case of aerobic growth of sphingomonads, oxidation is the favored mechanism of phenathrene degradation (Waigi et al., 2015). As with other PAHs, aerobic degradation of phenanthrene is initiated by the addition of two oxygen atoms to the aromatic ring, an event that is catalyzed by an aromatic-ring dioxygenase (Figure 5A). The generated *cis-*dihydrodiol molecules are subsequently dehydrogenated and cleaved by dehydrogenase and dioxygenase enzymes, respectively, to form the intermediate metabolite 1-hydroxy-2-naphtoic acid. This metabolite can be further degraded by two different routes: it can be processed by dioxygenases and dehydrogenases (*ortho*-cleavage route) to generate *o*-phtalate, which is later metabolized through protocatechuate to generate tricarboxylic acid (TCA) intermediates (Figure 5, steps 8A to 16A). Alternatively, by the *meta*-cleavage route, 1-hydroxy-2-naphtoic acid is decarboxylated to generate 1,2-dihydroxinaphtalene, which is further serially processed by dioxygenase, isomerase, aldolase, and dehydrogenase activities to produce salicylic acid (Figure 5, steps 8B to 12B; Goyal and Zylstra, 1997; Waigi et al., 2015). Salicylic acid is then further processed through gentisate (steps 13C to 16C) or catechol pathways (steps 13B to 18B), to generate TCA cycle intermediates, with the catechol pathway generating the yellow colored hydroximuconic acid used for the qualitative selection of strains in our screening approach.

To understand the mechanisms involved in phenanthrene degradation by *S. xenophagum* D43FB, we proceeded to do whole genome sequencing (Table S2), and based on putative gene function assignation, analyzed the possible PAH degradation pathways. As seen in Figure 5B, several operons are encoded in the D43FB genome that encode functions required for PAH degradation, including several genes encoding aromatic ring dioxygenases. The two biggest operons, encompassing all genes encoded in between *SxD43FB_20905* and *SxD43FB_21000* ORFs, are organized in opposing fashion, resembling the structure of previously described operons of *Sphingobium yanoikuyae* involved in the biodegradation of several PAH including napthalene and phenanthrene (Kim and Zylstra, 1999; Schuler et al., 2009). Also, similarly to the structure of *S. yanoikuyae* operons, and differing from PAH degradation operon structure in *Pseudomonas* species (where all genes for degradation of PAHs into aromatic acids are encoded in one “upper pathway” operon, and genes for the conversion of aromatic acids into TCA cycle intermediates are arranged in a 'lower pathway' operon; Li et al., 2004), PAH degrading genes in *S. xenophagum* D43FB operons are not ordered in their sequential enzymatic reactions. Rather, gene functions for “upper” and “lower” pathways are encoded in all encountered PAH degrading operons. After assigning gene functions to their corresponding degradation pathways (Figure 5A), most aerobic degradation steps can putatively be performed by *S. xenophagum* D43FB, except for the first steps of the *ortho*-cleavage route, were several enzymes seem to be missing (or alternatively, the genes encoding these functions could diverge from the prototypical described PAH enzyme encoding genes). No homologs for *nmsABC* or *bnsABCDEFGH* genes, involved in anaerobic degradation of PAHs, were found (Meckenstock et al., 2016). These results correlate with our inability to generate anaerobic growth of D43FB in the presence of phenanthrene as sole energy source (results not shown).

We analyzed the D43FB genome for other genes to support our previous experimental data (Table S2). Correlated with biofilm formation, *Sphingomonas* strains are known to produce and secrete a variety of extracellular polysaccharides (Thorne et al., 2000). Similarity analysis of the D43FB genome revealed the presence of genes related with bacterial cellulose production. Also, several genes encoding factors involved in stress response and cadmium and heavy metal resistance were found. A set of genes encoding Cd-transporters were determined in the genome of D43FB, a result that agrees with the high Cd^2+^ resistance observed in this strain. Finally, although D43FB failed to show chemotactic responses, several genes encoding components of the flagellar system were found, suggesting that this strain has the potential to swim and respond to attractants in its environments, under the proper gene inducing conditions. Altogether, these results suggest that the D43FB genome harbors a richness of genes involved in PAH metabolism and heavy metal resistance, supporting the high bioremediation potential of this strain.

## Discussion

Microbial bioremediation is quickly becoming an important approach in the continuing efforts to decontaminate critical sites of oil spillage in cold weather environments (Yang et al., 2009; Camenzuli and Freidman, 2015; Dias et al., 2015). Antarctica is a region in which the introduction of foreign organisms is forbidden, hence a good understanding of the available native bacteria available to bioremediate contaminated Antarctic areas, as well as a good comprehension of their metabolizing power and the mechanisms underlying PAH degradation will be key in the development of more efficient decontamination strategies. In this work, we have developed a simple and quick three-step enrichment and screening process that led us to the discovery of three phenanthrene degrading strains present in diesel contaminated Antarctic soils, a *Sphingobium* strain, a *Pseudomonas* strain and a *Rhodococcus* strain. These three bacterial genera have been previously described to degrade a variety of compounds, including PAHs (Aislabie et al., 2006). One isolate in particular, *S. xenophagum* D43FB, showed the ability to replicate at fast rates using both phenanthrene or diesel as sole carbon sources, and its able to degrade up to 95% of initial phenanthrene concentrations, following similar kinetics of growth and degradation to other high efficiency PAH degrading strains isolated by other research groups (Moody et al., 2001; Nadalig et al., 2002; Kuppusamy et al., 2016).

Antarctic soils exhibit a range of bacterial diversities, with the abundance of representative phyla changing depending on the properties of the soil, like the nutrient and moisture content, with distinct bacterial community profiles of varied heterogeneity exhibited in different microenvironments (Clocksin et al., 2007; Cary et al., 2010; Pudasaini et al., 2017). Bacteria from Cyanobacteria, Acidobacteria, Actinobacteria, Chloroflexi, and Proteobacteria phyla usually compose these heterogeneous communities. The presence of xenobiotics, such as PAHs, promote dramatic changes in the profile of the autochthonous soil community, decreasing biodiversity, and increasing the abundance of *Achromobacter, Rhodoccocus, Burkholderia, Pseudomonas*, and *Sphingomonas* species (van Dorst et al., 2016). This correlates with the isolation of potent *Rhodococcus, Sphingomonas*, and *Pseudomonas* PAH degrading strains in this work. Several PAH degrading strains of the *Sphingomonadaceae* family have been isolated from soils from the southern end of the Argentinian patagonia (Madueño et al., 2011), ratifying the presence of this type of PAH degrading bacteria in cold environment soils. Interestingly, other *S. xenophagum* strains reported to date correspond to PAH degrading isolates from Arctic or near-Arctic origin (Nohynek et al., 1996; Thomassin-Lacroix et al., 2001). *P. guineae* and *R. erythropolis* strains have also been isolated at Antarctic sites, with the latter one also being isolated from oil-contaminated soils (Whyte et al., 2002; Bozal et al., 2007). Our bacterial isolation approach was based on the enrichment of phenanthrene degrading strains from soil samples, and the screening of these strains for the most potent PAH metabolizers. Although, we have successfully isolated several bacteria of interest, it exists the possibility that other strains of interest in our soil samples were not identified, as our focus was biased toward strains that could be readily grown in standard laboratory conditions. Novel techniques of *in situ* microculturing have been used to culture Antarctic soil bacteria, by means of the soil substrate membrane system (SSMS), which recreates the native conditions of growth by using the collected soil samples as the nutrient source. SSMS culture has shown to retrieve a more diverse array of bacteria from soil samples, and has successfully been used to isolate PAH degrading bacteria (van Dorst et al., 2016; Pudasaini et al., 2017). Future approaches combining more representative culture techniques with our screening methods will possibly help to isolate a wider variety of potent PAH degrading strains.

Varied processes have been described to enhance the ability of bacteria to metabolize PAHs, including the formation of biofilms, secretion of biosurfactants, and the ability to perform chemotaxis. While *S. xenophagum* D43FB was unable to produce biosurfactants and did not exhibit chemotactic responses, this strain exhibited the ability to form robust biofilms *in vitro* and was able to adhere directly to phenanthrene crystals, as shown by SEM microscopy, suggesting this bacterial isolate can tightly interact with phenanthrene when using it as its main carbon source. Additionally, genomic analysis of D43FB revealed the presence of several genes encoding dioxygenase proteins, required for the processes of ring-cleavage required for aerobic degradation of PAHs, supporting the notion this strain can degrade aromatic molecules and generate degradation products that can be directed toward energy-producing metabolic pathways.

This work highlights how a simple screening process used with samples taken from extreme environments can uncover bacterial isolates of powerful metabolic properties. Future characterization of the isolated strains presented in this work, to understand their ability to degrade complex multi-ring PAH compounds in bioaugmentation experiments, as well as their effect over the dynamics of the endemic bacterial soil communities, will help to the development of efficient strategies for the *in situ* bioremediation of oil contaminated sites in the south pole and cold environments.

## Author contributions

AG, EF, and JP conceived and designed the study. AG performed the experiments. JJ performed bacterial identifications and analyzed the genomic data. AG, JJ, EF, DB, and JP analyzed the data and prepared the manuscript.

### Conflict of interest statement

DB, JJ, and JP are currently affiliated with uBiome, a private company which produces microbiome related products. In no way has their affiliation to uBiome influenced or shaped the experiments and the conclusions of this article. The other authors declare that the research was conducted in the absence of any commercial or financial relationships that could be construed as a potential conflict of interest.
