# Supplementary material for: Isolation and Characterization of Phenanthrene Degrading Bacteria from Diesel Fuel-Contaminated Antarctic Soils
Source: Front Microbiol. 2017 Aug 28;8:1634. doi: 10.3389/fmicb.2017.01634 (PMC5581505; doi:10.3389/fmicb.2017.01634)
Supplement: Supplementary file 1 [file DataSheet1.docx]

**TABLE S1**

|  | ***R. erythropolis* D32AFA** | ***S. xenophagum* D43FB** | ***P. guineae* E43FB** |
| --- | --- | --- | --- |
| *Enzymatic activity* |  |  |  |
| Nitrate reduction | + | − | − |
| Tryptophanase | + | + | + |
| Glucose fermentation | − | − | − |
| Arginine dihydrolase | − | − | − |
| Urease | − | − | − |
| β glucosidase | + | + | + |
| Gelatinase | + | − | − |
| β galactosidase | + | + | + |
|  |  |  |  |
| *C-source for growth* |  |  |  |
| D-glucose | + | + | + |
| L-arabinose | + | + | + |
| D-mannose | + | + | − |
| D-mannitol | + | + | − |
| N-acetyl-glucosamine | + | − | − |
| D-maltose | + | + | + |
| Potassium gluconate | + | +/− | − |
| Capric acid | + | + | − |
| Adipic acid | + | − | − |
| Malic acid | + | + | − |
| Phenyl acetic acid | + | + | − |
| Citrate trisodium | + | + | − |
| Cytochrome oxidase | − | + | + |

**TABLE S2**

|  |  |  |  |
| --- | --- | --- | --- |
|  |  |  |  |
|  | **Gene number** | **Putative encoded function** |  |
|  |  |  |  |
|  | *PAH and aromatic acid metabolism* | |  |
|  | SxD43FB_12035 | Maeylpyruvate isomerase |  |
|  | SxD43FB_12045 | Homogentisate 1,2-dioxygenase |  |
|  | SxD43FB_14215 | 4-carboxy-2-hydroxymuconate-6-semialdehyde dehydrogenase |  |
|  | SxD43FB_14220 | Protocatechuate 4,5-dioxygenase beta chain |  |
|  | SxD43FB_14225 | Protocatechuate 4,5-dioxygenase alpha chain |  |
|  | SxD43FB_14230 | 4-oxalomesaconate hydratase |  |
|  | SxD43FB_14245 | 4-hydroxy-4-methyl-2-oxoglutarate aldolase |  |
|  | SxD43FB_14250 | 4-oxalomesaconate tautomerase |  |
|  | SxD43FB_14255 | 2-pyrone-4,6-dicarbaxylate hydrolase |  |
|  | SxD43FB_20245 | Terephthalate 1,2-dioxygenase terminal oxygenase component subunite beta 2 |  |
|  | SxD43FB_20250 | Salicylate-5-hydroxylase large oxygenase component |  |
|  | SxD43FB_20255 | Trans-O-hydroxybenzylidenepyruvate hydratase-aldolase |  |
|  | SxD43FB_20905 | Naphthalene 1,2-dioxygenase subunit alpha |  |
|  | SxD43FB_20910 | Naphthalene 1,2-dioxygenase subunit beta |  |
|  | SxD43FB_20915 | Benzene 1,2-dioxygenase subunit alpha |  |
|  | SxD43FB_20920 | Biphenil 2,3-dioxygenase subunit beta |  |
|  | SxD43FB_20925 | Xylene 1-monooxygenase 1 |  |
|  | SxD43FB_20930 | Xylene monooxygenase electron transfer component (ferredoxin subunit) |  |
|  | SxD43FB_20940 | Cis-2,3-dihydrobiphenyl-2,3-diol dehydrogenase |  |
|  | SxD43FB_20945 | Aldehyde dehydrogenase |  |
|  | SxD43FB_20955 | 2-hydroxymuconate tautomerase |  |
|  | SxD43FB_20960 | 4-oxalocrotonate decarboxylase |  |
|  | SxD43FB_20965 | 4-hydroxy-2-oxovalerate aldolase |  |
|  | SxD43FB_20970 | Acetaldehyde dehydrogenase |  |
|  | SxD43FB_20975 | 2-oxopent-4-enoate hydratase |  |
|  | SxD43FB_20980 | 2-hydroxymuconic semialdehyde dehydrogenase |  |
|  | SxD43FB_20990 | 2-hydroxymuconic semialdehyde dehydrogenase |  |
|  | SxD43FB_21000 | Catechol 2,3-dioxygenase |  |
|  | SxD43FB_21350 | 2-hydroxymuconate semialdehyde hydrolase |  |
|  | SxD43FB_21525 | Cis-2,3-dihydrobiphenyl-2,3-diol dehydrogenase |  |
|  | SxD43FB_21530 | Aldehyde dehydrogenase |  |
|  | SxD43FB_21535 | Ferredoxin-1 subunit |  |
|  | SxD43FB_21540 | 2-hydroxymuconate tautomerase |  |
|  | SxD43FB_21545 | 4-oxalocrotonate decarboxylase |  |
|  | SxD43FB_21550 | 4-hydroxy-2-oxovalerate aldolase |  |
|  | SxD43FB_21555 | Acetaldehyde dehydrogenase |  |
|  | SxD43FB_21560 | 2-keto-4-pentenoate hydratase |  |
|  | SxD43FB_21565 | 2-hydroxymuconic semialdehyde dehydrogenase |  |
|  | SxD43FB_21575 | Catechol 2,3-dioxygenase |  |
|  | SxD43FB_21580 | 2-hydroxymuconate semialdehyde hydrolase |  |
|  | SxD43FB_21585 | Glutathione S-transferase |  |
|  | SxD43FB_21590 | Anthranilate 1,2-dioxygenase small subunit |  |
|  | SxD43FB_21595 | Anthranilate 1,2-dioxygenase large subunit |  |
|  | SxD43FB_22195 | 2-hydroxychromene-2-carboxylate isomerase |  |
|  | SxD43FB_22200 | Anthranilate 1,2-dioxygenase large subunit |  |
|  | SxD43FB_22205 | Anthranilate 1,2-dioxygenase small subunit |  |
|  | SxD43FB_22210 | 1,2-dihydroxynaphthalene dioxygenase ferredoxin subunit |  |
|  | SxD43FB_22215 | 1,2-dihydroxynaphthalene dioxygenase |  |
|  |  |  |  |
|  | *Stress response* |  |  |
|  | SxD43FB_07400 | Exopolyphosphatase, *ppx* |  |
|  | SxD43FB_02155 | Polyphosphate kinase, *ppk* |  |
|  | SxD43FB_02150 | pppGpp pyrophosphatase, *gppA* |  |
|  |  |  |  |
|  | *Heavy metal resistance* |  |  |
|  | SxD43FB_02870 | Cadmium / Cobalt / Zinc /H(+)-K(+) antiporter |  |
|  | SxD43FB_07040 | Cobalt / Zinc / Cadmium resistance protein |  |
|  | SxD43FB_07045 | Cobalt / Zinc / Cadmium resistance protein |  |
|  | SxD43FB_07050 | Cobalt / Nickel resistance protein |  |
|  | SxD43FB_07070 | Zinc transporter |  |
|  | SxD43FB_07075 | Cadmium transport ATPase |  |
|  | SxD43FB_07335 | Cobalt / Zinc / Cadmium resistance protein |  |
|  | SxD43FB_07330 | Cation / heavy metal efflux protein |  |
|  | SxD43FB_07325 | Cation / heavy metal efflux protein |  |
|  | SxD43FB_10610 | Cobalt / Zinc / Cadmium resistance protein |  |
|  | SxD43FB_10615 | Cobalt / Zinc / Cadmium resistance protein |  |
|  | SxD43FB_10620 | Cobalt / Nickel resistance protein |  |
|  | SxD43FB_10625 | Cadmium transport ATPase |  |
|  | SxD43FB_11315 | ATM1 type heavy metal exporter |  |
|  | SxD43FB_18465 | ATM1 type heavy metal exporter |  |
|  |  |  |  |
|  | *Biofilm formation* |  |  |
|  | SxD43FB_19035 | Cellulose synthase protein *acsAB* |  |
|  | SxD43FB_19030 | Cellulose synthase protein *acsC* |  |
|  | SxD43FB_20060 | Cellulose synthase protein *bcsA* |  |
|  |  |  |  |
|  | *Chemotaxis* |  |  |
|  | SxD43FB_16930 | Flagellar motor switch protein *fliM* |  |
|  | SxD43FB_16925 | Flagellar motor switch protein *fliN* |  |
|  | SxD43FB_16915 | Flagellar biosynthetic protein *fliP* |  |
|  | SxD43FB_16910 | Flagellar biosynthetic protein *fliQ* |  |
|  | SxD43FB_16905 | Flagellar biosynthetic protein *fliR* |  |
|  | SxD43FB_16900 | Flagellar biosynthetic protein *flhB* |  |
|  | SxD43FB_16885 | Flagellar hook-associated protein *fliD* |  |
|  | SxD43FB_16880 | Flagellar protein *fliS* |  |
|  |  |  |  |
|  |  |  |  |
|  |  |  |  |
